# A new species of *Fannia* (Diptera, Fanniidae) from Yunnan, China

**DOI:** 10.3897/zookeys.862.34280

**Published:** 2019-07-09

**Authors:** Li-ping Yan, Wen-tian Xu, Ming-fu Wang, Dong Zhang

**Affiliations:** 1 College of Nature Conservation, Beijing Forestry University, Beijing 100083, China Beijing Forestry University Beijing China; 2 Institute of Entomology, Shenyang Normal University, Shenyang 110034, China Shenyang Normal University Shenyang China

**Keywords:** Description, *Fanniaposticata*-group, male terminalia

## Abstract

A new species of the genus *Fannia* (Diptera, Fanniidae) is described from Yunnan, China, namely *Fanniabaihualingensis***sp. nov.** The male habitus as well as terminalia are documented with focus-stacked photographs. A detailed comparison of new species with related species is provided.

## Introduction

The Fanniidae (Diptera, Muscoidea) are cosmopolitan flies with over 400 described species. Around 160 species have been found from China, including one species of *Euryomma* Stein, two species of *Piezura* Rondani, and 157 species of *Fannia* Robineu-Desvoidy. Of these species, 61.25% of them (i.e., 98 species) are endemic to China.

The *Fanniaposticata*-group was established by Chillcott (1961), originally as the *Fanniapretiosa*-group. Wang et al. (2010) reviewed the *F.posticata*-group and expanded it to include 21 species.

Yunnan is the highest biodiversity hotspot in China. In this study, we describe a new species of the *Fanniaposticata*-group from Yunnan, *Fanniabaihualingensis* sp. nov., and provide an extensive documentation of the adult male of this species.

## Materials and methods

Terminology follows McAlpine (1981) and Stuckenberg (1999). Methods for the preparation of terminalia and illustrations follow Zhang et al. (2013). All type specimens of the new species are deposited in the Museum of Beijing Forestry University, Beijing, China (MBFU).

Abbreviations used throughout the text are as follows:

acr acrostichal seta,

ad anterodorsal seta,

av anteroventral seta,

d dorsal seta,

p posterior seta,

pd posterodorsal seta,

pv posteroventral seta.

## Taxonomy

### Fannia baihualingensis sp. nov.

Taxon classificationAnimaliaDipteraFanniidae

http://zoobank.org/DA934C3A-C351-424A-8421-30EC259742D1

Figures 1 , 2

#### Material examined.

Holotype ♂: China: Yunnan, Gaoligong, Baihualing, 25.VII.2015, Coll. L.P. Yan & C. Wang (MBFU).

Paratypes 2 ♂, same data as holotype (MBFU).

#### Diagnosis.

*Fanniabaihualingensis* can be readily identified by the following character states: distinctly projecting lower calypter; hind coxa bare on posterior surface; hind femur arcuate, with clump of long black setae on swollen part; hind tibia with two av, hook-like projection on lower margin of cercus curved outward; surstylus very long and slender; bacilliform process absent.

#### Description.

Male. Body length 5.00–6.50 mm (2 specimens measured). Eye bare. Fronto-orbital plate and parafacial with grayish-silvery pollinosity. Frons slightly narrower than the distance between two posterior ocelli at narrowest point, frontal vitta black, frontal setae 7–9, stout. Postocular setae in 1 row, without occipital seta behind the postocular setae on vertex. Parafacial bare, at middle about 3/4 as wide as the width of postpedicel. Antenna grayish black, postpedicel 2 × longer than wide, arista black and short plumose, slightly swollen in basal part. Epistoma not projecting beyond vibrissal angle, vibrissal angle behind frontal angle in profile, subvibrissal setae in 1 row, lateral with one 1 of short setae. Proboscis stout. Palpus black, claviform, longer than the length of prementum.

**Figure 1.**
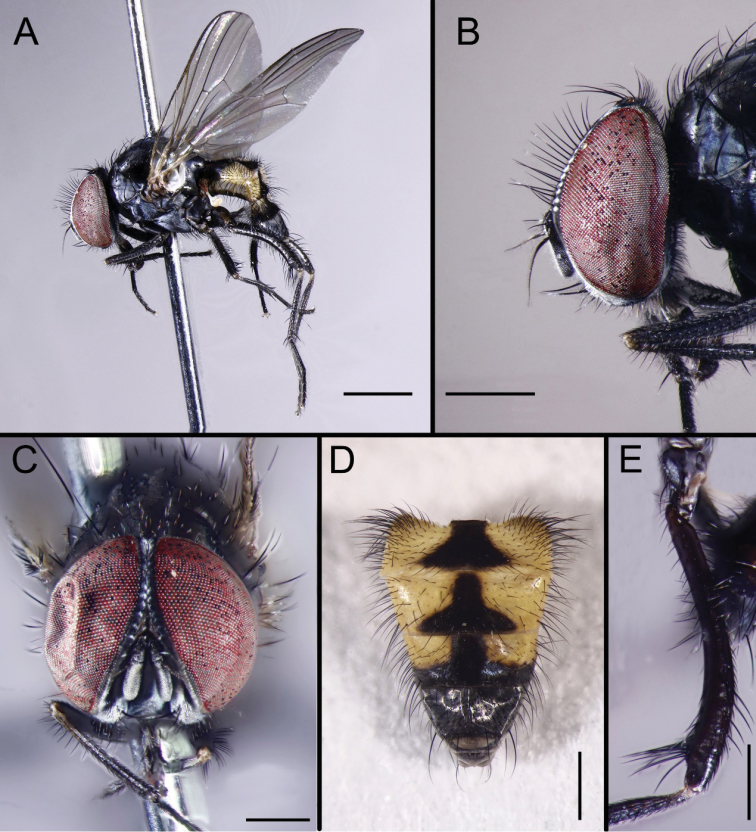
*Fanniabaihualingensis* sp. nov. from Yunnan, China, male. **A** Habitus, lateral view **B** Head, lateral view **C** Head, anterior view **D** Abdomen, dorsal view **E** hind femur, anterior view. Scale bars: 1.00 mm (**A**); 0.50 mm (**B–E**).

Thorax ground color black, without distinct vitta. Postpronotal lobe gray. Presutural acr biserial, hair-like, only prescutellar pairs slightly stout, dorsocentrals 2+3, intra-alars 2, supra-alars 2, postpronotals 2, notopleurals 2. Katepisternal setae 1+1, katepisternum without ventral spine. Scutellum black, with 3 pairs of lateral, 2 pair of discal, and 2 pair of apical setae. Calypters white, the lower one slightly projecting beyond the upper one. Wing brownish; veins brown; tegula dark brown; basicosta brownish-yellow; costal spine inconspicuous; node of Rs bare on ventral and dorsal surfaces; vein R_4+5_ straight; crossveins without obvious cloud; haltere yellow but brown in basal part. Legs entirely black. Fore femur with complete d, pd, and pv rows, fore tibia with 1 pd seta; mid coxa with spin-like setae, mid femur with complete ad row, becoming gradually shorter and denser towards apex, pv row complete, in 1 row, ad rows weak, 5 setae strong in distal part, mid tibia slightly swollen in distal 2/3, with 1 ad and 1 pd; hind coxa bare on posterior surface, hind femur curved and arcuate, swollen at apex below, the swollen part with a clump of long black setae on postero-ventral (Fig. 1E), antero-ventral surface with a complete series of setae, (the apical 4 longer and stronger), hind tibia with 2 av.

**Figure 2.**
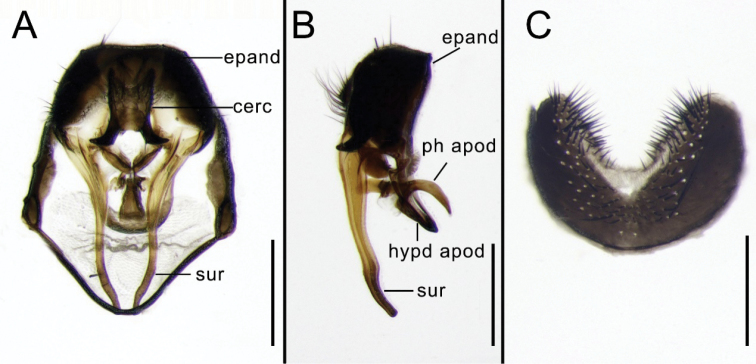
*Fanniabaihualingensis* sp. nov. from Yunnan, China, male. **A** Terminalia, ventral view **B** Terminalia, lateral view **C** Sternite 5, ventral view. Abbreviations: cerc = cercus; epand = epandrium; hypd apod = hypandrial apodeme; ph apod = phallapodeme; sur = surstylus. Scale bar: 0.25 mm.

Abdomen long, depressed and flattened. Syntergite 1+2 dark in basal part. Syntergite 1+2 to tergite 4 largely yellow with 1 median inverted black triangular vitta (Fig. 1D). Distal half of tergite 4 and all of tergite 5 black gray-pollinose, each tergite with long lateral marginal setae. Sternite 5 profoundly indented on posterior margin and covered with setae (Fig. 2C). Cercus slightly rounded, the hook-like projection on its lower margin curved outward. Surstylus very long and slender (Fig. 2A), slightly arcuate on apical half. Bacilliform process absent.

Female: Unknown.

#### Remarks.

According to the keys by Chillcott (1961), Hennig (1955), and Wang et al. (2010) and the detailed description by Chillcott (1961: 142), Pont (1977: 19), and Hennig (1955:42), *F.baihualingensis* sp. nov. resembles *F.arcuata*, *F.curvipes*, *F.fasciculata*, *F.gilvitarsis*, and *F.anteroventralis* due to the strongly arcuate hind femur. *Fanniagilvitarsis* and *F.anteroventralis* has a black ground-color. The abdomen of *F.baihualingensis* sp. nov. is yellow in lateral part. *Fanniaanteroventralis* has a black haltere at apex, *F.baihualingensis* sp. nov. has a yellow haltere at apex.

**Figure 3.**
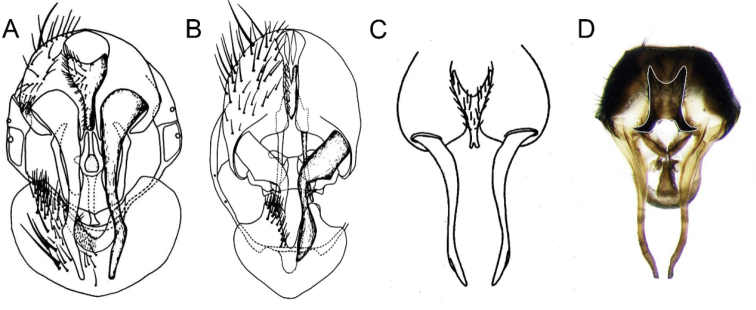
Male terminalia of *Fannia* spp. **A***F.curvipes* Malloch (adapted from Chillcott 1961: fig. 88) **B** Male terminalia, *F.arcuata* Chillcott (adapted from Chillcott 1961: fig. 89) **C** Male terminalia, *F.fasciculata* (Loew) (adapted from Hennig 1955: pl. 4, fig. 75) **D** Male terminalia, *Fanniabaihualingensis* sp. nov.

Compared with *F.arcuata*, *F.curvipes*, and *F.fasciculata*, only the hind tibia of *F.arcuata* has a complete ad row. *Fanniacurvipes*, *F.fasciculate*, and *Fanniabaihualingensis* sp. nov. are all only with one ad. The male terminalia of *F.arcuata* is also very different from those of the other three species (Fig. 3B).

The male terminalia of *F.curvipes*, *F.fasciculate*, and *Fanniabaihualingensis* sp. nov. are very similar, especially the shape of surstylus. However, the new species can be identified by the shape of the cercus. In *F.curvipes* the cercal plate is very slender in its apical half and prolonged into an upcurved process (Fig. 3A). The cercus of *F.fasciculata* is slender and bifurcate at the apex (Fig. 3C). The cercus of *F.baihualingensis* sp. nov. is slightly rounded, with the hook-like projection on its lower margin strongly curved outward (Fig. 3D).

*Fanniabaihualingensis* sp. nov. can also be distinguished from *F.fasciculata* by some external characters, such as the number of av on hind tibia: the new species has only two av, while *F.fasciculata* has four or five.

#### Etymology.

The new species is named after its type locality, Baihualing.

#### Distribution.

Known only from the type locality in Yunnan, China.

## Supplementary Material

XML Treatment for Fannia baihualingensis

## References

[B1] Chillcott JG (1961) A revision of the Neartic species of Fanniinae (Diptera: Muscidae). Canadian Entomologist, Supplement 14(1960): 1–295. 10.4039/entm9214fv

[B2] Hennig W (1955–1964) : Family Muscidae. In: Lindner E (Ed.) Die Fliegen der Paläarktischen Region 63b (part).Schweizerbart, Stuttgart, 1–99.

[B3] McAlpine JF (1981) Morphology and terminology – adults. In: McAlpine JF Peterson BV Shewell GE Teskey HJ Vockeroth JR Wood DM (Eds) Manual of Nearctic Diptera. Vol. 1.Research Branch Agriculture Canada Monograph 27: 9–63.

[B4] Pont AC (1977) A revision of Australian Fanniidae (Diptera: Calyptratae). Australian Journal of Zoology, Supplementary Series 51: 1–60. 10.1071/AJZS051

[B5] Stuckenberg BR (1999) Antennal evolution in the Brachycera (Diptera), with a reassessment of terminology relating to the flagellum. Studia Dipterologica 6: 33–48.

[B6] Wang MF Zhang D Cheng XL (2010) Taxonomic review of the *posticata*-group of *Fannia* Robineau-Desvoidy (Diptera: Fanniidae), with the description of two new species from China. Annales de la Société entomologique de France (N.S.) 46: 481–485. 10.1080/00379271.2010.10697685

[B7] Zhang D Zhang M Pape T Gu CW Wu W (2013) Sarcophaga (Hoa) flexuosa Ho (Diptera: Sarcophagidae): association of sexes using morphological and molecular approaches, and a re-definition of *Hoa* Rohdendorf. Zootaxa 3670: 71–79. 26438923

